# Sleep Quality and Cognitive Performance in Chronic Primary Musculoskeletal Pain: An Observational Study With Healthy Controls

**DOI:** 10.1002/ejp.70085

**Published:** 2025-07-24

**Authors:** Alejandro Arévalo‐Martínez, Carlos Barbosa‐Torres, María Elena García‐Baamonde, César Luis Díaz‐Muñoz, Juan Manuel Moreno‐Manso

**Affiliations:** ^1^ Department of Psychology, Faculty of Education and Psychology University of Extremadura Badajoz Spain; ^2^ Department of Medical‐Surgical Therapy, Faculty of Medicine University of Extremadura Badajoz Spain

**Keywords:** chronic pain, cognitive performance, executive functions, neuropsychological assessment, sleep quality

## Abstract

**Background:**

Chronic primary musculoskeletal pain (CPMP) is a recently defined diagnosis characterised by persistent pain accompanied by emotional distress and/or functional disability. Although sleep disturbances and cognitive complaints are common in individuals with chronic pain, few studies have examined their relationship in CPMP, a condition that remains underexplored. This study aimed to examine whether cognitive functioning in individuals with CPMP is affected by sleep quality, exploring its potential contribution across cognitive domains to the neuropsychological profile of this condition.

**Methods:**

This cross‐sectional study included 60 adults, of whom 30 were diagnosed with CPMP and 30 were healthy controls. Participants completed the Numeric Pain Rating Scale, the Symptom Checklist‐90‐Revised and the Pittsburgh Sleep Quality Index, followed by a standardised neuropsychological assessment consisting of the Stroop test, the TESEN, the Rings Test and the Working Memory Index from the Wechsler Adult Intelligence Scale.

**Results:**

Patients with chronic pain exhibited mild cognitive impairment in selective and sustained attention, processing speed, working memory, planning, problem solving, inhibition and resistance to interference. Poorer sleep quality was independently associated with greater cognitive impairment, particularly in sustained attention, processing speed and working memory. Sleep quality significantly predicted performance in these cognitive domains, beyond the influence of sociodemographic factors, pain intensity and psychological distress.

**Conclusions:**

This study contributes to characterising the neuropsychological profile of individuals with CPMP and highlights the role of sleep quality in cognitive functioning. These findings may help inform interventions aimed at improving executive performance in chronic pain populations.

**Significance Statement:**

This study contributes to a deeper understanding of the cognitive difficulties experienced by individuals with chronic primary musculoskeletal pain, a recently defined diagnosis in the 11th revision of the International Classification of Diseases. It highlights the importance of sleep quality as a predictor of cognitive performance—particularly in attention, processing speed and working memory—independently of pain intensity and psychological distress. These findings support the integration of sleep‐focused strategies into assessment and intervention approaches in chronic pain populations.

## 1 Introduction

Sleep plays a fundamental role in regulating multiple physiological, emotional, and cognitive processes. Beyond being a basic biological necessity, it is essential for maintaining neuronal homeostasis, consolidating memory and restoring executive functioning (Goldstein and Walker 2014; Jain et al. 2024; Scullin and Bliwise 2015). The optimal sleep duration ranges between 7 and 8 h per day, and deviations from this interval—whether shorter or longer—have been linked to adverse effects on both physical and mental health (Kong et al. 2023; Xu et al. 2020). Globally, sleep‐related problems are widespread, with prevalence rates between 3.9% and 45% in the adult population and up to 70% in older adults (Canever et al. 2024; Wang et al. 2023). The most common disturbances include poor sleep quality, sleep apnoea, insomnia and excessive daytime sleepiness (Canever et al. 2024).

In the context of chronic diseases, the scientific literature has identified sleep‐related difficulties as a frequent problem among individuals with chronic musculoskeletal pain (Goossens et al. 2025; Jain et al. 2024). Current theoretical models suggest that poor sleep quality may amplify the impact of pain on neuropsychological functioning, leading to cognitive impairment (BlaisdaleJones et al. 2021; Seiger et al. 2024). Such impairment may hinder pain coping strategies and increase psychological distress, thereby intensifying the pain experience and reinforcing the interaction between pain, cognitive dysfunction and emotional suffering (Finan et al. 2013; Seiger et al. 2024). Recent studies in patients with chronic musculoskeletal conditions, such as low back pain or fibromyalgia, support these models, showing that poor sleep quality is associated with greater cognitive difficulties (Jain et al. 2024; Whale and Gooberman‐Hill 2022). These findings suggest that altered sleep patterns should not only be seen as a consequence of chronic pain, but also as a key modulator of its impact on cognitive function, with sleep quality emerging as a particularly relevant factor (Scullin and Bliwise 2015; Seiger et al. 2024; Xu et al. 2020).

In this regard, the latest revision of the International Classification of Diseases (ICD‐11; World Health Organization 2018) introduced a new diagnosis, chronic primary musculoskeletal pain (CPMP). This condition is characterised by persistent pain accompanied by significant emotional distress and/or functional disability, in the absence of a medical condition that adequately explains the symptoms (World Health Organization 2018). Although research on CPMP is still limited, this diagnosis has been consistently associated with generalised cognitive deficits (Cohen et al. 2021). These deficits cannot be explained solely by pain or the somatic, depressive or anxious symptoms commonly present in this population; rather, they also appear to be mediated by sleep quality, reinforcing the need to consider this factor when evaluating both clinical progression and its impact on daily cognitive functioning (Cohen et al. 2021; Fang et al. 2019; Finan et al. 2013).

Based on the above reasoning, this study aimed to explore whether cognitive functioning in individuals with chronic primary musculoskeletal pain (CPMP) is affected by sleep quality, independently of pain intensity, psychological distress and sociodemographic characteristics. Specifically, it seeks to compare differences in cognitive functioning between individuals with CPMP and healthy controls, examine the association between sleep quality and cognitive performance within the CPMP group and determine whether sleep quality may predict performance across different cognitive domains.

## 2 Method

### 2.1 Participants

This observational study employed a cross‐sectional design and included a total of 60 individuals, with 30 patients diagnosed with chronic primary musculoskeletal pain (CPMP), according to the criteria set out in the International Classification of Diseases, 11th Revision (ICD‐11; World Health Organization 2018), and 30 healthy participants who comprised the control group. A nonprobabilistic sampling method was used for recruitment.

Patients were selected from a specialised rehabilitation centre focused on the comprehensive care of individuals with physical and neurological conditions and had received a diagnosis of CPMP prior to their participation. The centre is part of the Regional Health Service of Extremadura, Spain. Eligible participants were between 18 and 80 years of age—an age range chosen based on epidemiological studies indicating a higher prevalence of chronic musculoskeletal pain among older adults (de Souza et al. 2019; Wong et al. 2022). As part of the initial assessment, the diagnosis was confirmed using a structured interview. Exclusion criteria for the patient group included: experiencing pain for less than 3 months; having a medical, neurological or psychiatric condition that could better account for the symptoms; use of psychotropic or neurological medication, whether prescribed for chronic pain or for other conditions; or having received any form of physical or psychological treatment prior to participation.

The control group consisted of healthy individuals within the same age range, with no history of chronic pain, neurological or psychiatric disorders, and no current involvement in psychological treatment. These participants were recruited via community advertisements placed in local centres and online platforms.

All participants completed a set of questionnaires assessing demographic characteristics, followed by an individual neuropsychological evaluation. Information regarding pain intensity, pain duration and use of pain medication was collected exclusively in the CPMP group. To minimise potential biases related to age and educational differences, all instruments were standardised tests with normative data adjusted for both variables and validated in Spanish populations.

### 2.2 Instruments

1. The Numeric Pain Rating Scale (NPRS; Breivik et al. 2008). Pain intensity over the past 24 h was assessed using the NPRS, an 11‐point self‐report scale ranging from 0 ‘no pain’ to 10 (‘the worst pain imaginable’). Scores were categorised as follows: 0 = no pain, 1–3 = mild pain, 4–6 = moderate pain and 7–10 = severe pain (Breivik et al. 2008; Modarresi et al. 2022). This scale has shown good test–retest reliability in individuals with chronic pain (ICC = 0.58–0.96) and has also demonstrated good reliability in Spanish populations (Childs et al. 2005; Modarresi et al. 2021; Ubillos‐Landa et al. 2019; Vicente‐Herrero et al. 2018).
2. The Symptom Checklist‐90‐R (SCL‐90‐R; Derogatis 1994). Psychological distress was assessed using the SCL‐90‐R, a 90‐item self‐report questionnaire rated on a five‐point Likert scale (0 = not at all, 4 = extremely). The instrument measures nine primary symptom dimensions: somatisation, obsessive‐compulsive, interpersonal sensitivity, depression, anxiety, hostility, phobic anxiety, paranoid ideation and psychoticism. In addition, it provides three global indices: the Global Severity Index (GSI), the Positive Symptom Total (PST) and the Positive Symptom Distress Index (PSDI). In the present study, particular emphasis was placed on the GSI, which offers a comprehensive estimate of overall psychological distress by integrating both the number and intensity of symptoms reported. This decision was based on prior research in chronic illness populations supporting the GSI as a valid measure of global distress (Burris et al. 2010; Hardt et al. 2000; Yalachkov et al. 2019). T scores between 40 and 60 are considered within the normative range, whereas scores equal to or greater than 60 indicate clinically significant levels of distress. Regarding its psychometric properties, the Spanish version has shown good internal consistency (*α* = 0.77–0.90) (González de Rivera et al. 2002). In the present study, internal consistency was good for the full scale (*α* = 0.79) and high for the Global Severity Index (GSI; *α* = 0.83).
3. The Pittsburgh Sleep Quality Index (PSQI; Buysse et al. 1989). The PSQI is a 19‐item self‐report measure used to assess subjective sleep quality over the past month. Items are grouped into seven components: subjective sleep quality, sleep latency, sleep duration, habitual sleep efficiency, sleep disturbances, use of sleep medication and daytime dysfunction. The total score ranges from 0 to 21, with scores ≥ 5 indicating poor sleep quality. Regarding its psychometric properties, the Spanish version has shown good internal consistency (*α* = 0.81) (Royuela‐Rico and Macías‐Fernández 1997). In the present study, internal consistency was good (*α* = 0.75).
4. The Stroop Colour and Word Test (SCWT; Golden et al. 1978). The SCWT assesses inhibitory control, selective attention, processing speed and cognitive flexibility. It includes three conditions: word reading, colour naming and a colour‐word interference task. Performance is based on the number of correct responses within a time limit. T scores (*M* = 50; SD = 10) were used to interpret results as follows: ≤ 45 = below average performance, 46–55 = average performance and ≥ 56 = above average performance (Golden 2020). Regarding its psychometric properties, the Spanish version has shown good internal consistency (*α* = 0.85 for word, *α* = 0.81 for colour, *α* = 0.69 for word‐colour and *α* = 0.70 for interference) (Golden 2020). In the present study, internal consistency was good (*α* = 0.79 for word, *α* = 0.76 for colour, *α*= 0.75 for word‐colour and *α* = 0.72 for interference).
5. The Pathways Test (Test de los Senderos) (TESEN; Portellano and Martínez 2014). Based on the Trail Making Test (TMT, Reitan and Wolfson 1985), the TESEN assesses sustained and selective attention, visuomotor and visuospatial planning, cognitive flexibility, inhibition, working memory and prospective memory. It includes four paths of increasing difficulty. Scores reflect performance across three domains: execution, speed and accuracy. Decatypic scores (Mean = 5.5; SD = 2) were used to interpret results as follows: ≤ 4 = below average performance, 5–6 = average performance and ≥ 7 = above average performance. Regarding its psychometric properties, the TESEN, developed and standardised in Spain, has shown high internal consistency (*α* = 0.93 for speed, *α* = 0.89 for accuracy and *α* = 0.88 for execution) (Portellano and Martínez 2014). In the present study, internal consistency was high (*α* = 0.88 for speed, *α* = 0.84 for accuracy and *α* = 0.79 for execution).
6. The Rings Test (Test de las Anillas) (Portellano and Martínez 2011). Based on the Tower of Hanoi, this task evaluates planning, problem solving, sustained attention, working memory, cognitive flexibility, visuoconstructive abilities and visuomotor coordination. Participants rearrange coloured rings on three vertical pegs to replicate specific configurations across 15 trials. Performance is scored based on completion time and number of moves. Decatypic scores (Mean = 5.5; SD = 2) were used to interpret results as follows: ≤ 4 = below average performance, 5–6 = average performance and ≥ 7 = above average performance. Regarding its psychometric properties, the Rings Test, developed and standardised in Spain, has demonstrated high internal consistency (*α* = 0.92) (Portellano and Martínez 2011). In the present study, internal consistency was high (*α* = 0.84).
7. The Wechsler Adult Intelligence Scale 4th Edition—Working Memory Index (WAIS; Wechsler 2008). The Working Memory Index (WMI) of the WAIS‐IV comprises two subtests: Digit Span and Arithmetic. Digit Span requires repetition of numerical sequences in forward, backward and ascending order. Arithmetic involves solving orally presented math problems under time constraints. Composite scores (*M* = 100; SD = 15) were used to interpret results as follows: ≤ 89 = below average performance, 90–109 = average performance and ≥ 110 = above average performance. Regarding its psychometric properties, the Spanish version has shown high internal consistency (*α* = 0.92) (Wechsler 2012). In the present study, internal consistency was high (*α* = 0.82).

### 2.3 Procedure

This study was conducted in a clinic specialised in the integral rehabilitation of individuals with physical and neurological conditions. All procedures were approved by the Ethics Committee of the University of Extremadura (Ref.: 157/2023) and followed the principles outlined in the 1964 Declaration of Helsinki and its subsequent amendments or comparable ethical standards. Additionally, the study complied with the provisions of Spain's Organic Law 3/2018, of December 5th, on the Protection of Personal Data and Guarantee of Digital Rights, ensuring confidentiality and secure storage of all participant information.

The research team was composed of psychologists holding a Bachelor's degree in Psychology and a Master's degree in General Health Psychology. Prior to data collection, the researchers received training in the standardised administration of the instruments, including instructional sessions and detailed protocol review, to ensure procedural consistency and data reliability. The assessment session was conducted individually and lasted approximately 1 h per participant. All participants provided written informed consent prior to participation and did not receive any financial compensation. No comprehension difficulties were observed during the administration of the instruments.

### 2.4 Data Analysis

The data were analysed using IBM SPSS Statistics version 27 for Windows. Statistical significance was set at *p* < 0.05 for all analyses. Between‐group comparisons for continuous demographic and clinical variables were performed using Mann–Whitney's U test, given the nonparametric distribution of the data. Categorical variables were analysed using Pearson's *χ* ^2^ test with Yates' correction or Fisher's exact test, depending on the characteristics of the variable.

To compare cognitive performance between the CPMP group and the control group, Mann–Whitney's U tests were conducted for each of the cognitive outcome measures. To account for the increased risk of Type I errors due to multiple comparisons, the Benjamini–Hochberg procedure was applied to adjust *p* values and control the false discovery rate (FDR). Subsequently, partial correlation analyses using Spearman's rho were conducted to examine the associations between sleep quality and cognitive performance within the CPMP group, while controlling for age, sex, pain intensity and psychological distress. Finally, hierarchical linear regression analyses were carried out to determine whether sleep quality significantly predicted cognitive performance in the CPMP group, using the same covariates.

To evaluate the statistical power of our analyses, we conducted a post hoc power analysis. With a significance level of *α* = 0.05, we compared the SCWT performance between participants with CPMP and controls, obtaining adequate power (1 − *β* = 0.83). For the TESEN, the analysis yielded very high power (1 − *β* = 0.99). For the Rings Test, power was high (1 − *β* = 0.94), and a similar result was found for the WAIS–WMI (1 − *β* = 0.94). These results suggest that the study had sufficient power to detect the observed effects.

## 3 Results

The demographic and clinical characteristics of the participants are shown in Table 1. A total of 60 individuals were included in the study, with ages ranging from 41 to 75 years. The sample comprised 30 participants diagnosed with CPMP and 30 healthy controls. The mean age in the CPMP group was 57.60 years (SD = 8.51), while in the control group it was 54.13 years (SD = 5.82), with no statistically significant differences between the two groups (*p* = 0.087). The CPMP group included 22 women (73.3%), whereas the control group included 12 women (40%), indicating a significant difference in sex distribution (*p* = 0.019). No significant differences were found between groups in terms of civil status, education level or work situation (*p* > 0.05 for all comparisons). Regarding psychological distress and sleep quality, participants in the CPMP group scored significantly higher on both the GSI index of the SCL‐90‐R and the global score of the PSQI compared with the control group (*p* < 0.001 in both cases).

**TABLE 1:** Demographic and clinical characteristics of the CPMP and control groups.

	CPMP (*n* = 30)	CG (*n* = 30)	*p*
Age (mean years ± SD)	57.60 ± 8.51	54.13 ± 5.82	0.087^a^
Range (years)	41–75	44–69	
Sex, *n* (%)			
Male	8 (26.7)	18 (60)	0.019^b^
Female	22 (73.3)	12 (40)	
Civil status, *n* (%)			
Single	2 (6.7)	3 (10)	1^c^
Married	24 (80)	24 (80)	
Divorced	2 (6.7)	2 (6.7)	
Widowed	2 (6.7)	1 (3.3)	
Education, *n* (%)			
Primary	5 (16.7)	8 (26.7)	0.748^b^
Secondary	5 (16.7)	5 (16.7)	
Professional training	10 (33.3)	10 (33.3)	
University	10 (33.3)	7 (23.3)	
Work situation, *n* (%):			
Employed	20 (66.7)	24 (80)	0.164^c^
Unemployed	4 (13.3)	5 (16.7)	
Retired	6 (20)	1 (3.3)	
SCL‐90‐R—GSI Score (mean ± SD)	57.27 ± 9.54	40.97 6.69	< 0.001^a^
PSQI—Global score (mean ± SD)	11.17 ± 4.65	5.80 ± 3.32	< 0.001^a^
NPRS (mean ± SD)	5.23 ± 1.63	NA	NA
No pain	0	NA	NA
Mild	2	NA	NA
Moderate	23	NA	NA
Severe	5	NA	NA
Duration of pain (mean years ± SD)	11.95 ± 1.85	NA	NA
Range (years)	1–40	NA	NA
Medication for pain, *n* (%)			
Yes	26 (86.7)	NA	NA
No	4 (13.3)	NA	NA

*Note:* Values are expressed as mean ± standard deviation or *n* (%). ^a^ *p* value obtained using the Mann–Whitney U test; ^b^ *p* value obtained using Pearson's chi‐squared test; ^c^ *p* value obtained using Fisher's exact test.

Abbreviations: CG, control group; CPMP, chronic primary musculoskeletal pain; NA, not applicable; NPRS, Numeric Pain Rating Scale; PSQI, Pittsburgh Sleep Quality Index; SCL‐90‐R – GSI, Global Severity Index of the Symptom Checklist‐90‐R.

Participants in the CPMP group reported an average pain duration of 11.95 years (SD = 1.85). Regarding pain intensity, they showed a mean score of 5.23 (SD = 1.63) on the NPRS, indicating a moderate level of pain, with most participants falling within the moderate range (*n* = 23; 76.7%), and a smaller proportion reporting severe (*n* = 5; 16.7%) or mild pain (*n* = 2; 6.7%). In addition, 86.7% (*n* = 26) of participants reported taking prescribed pain medication, with paracetamol (31%), enantyum (24%) and ibuprofen (17%) being the most frequently used, followed by aceclofenac and diclofenac (14% each).

Group comparisons for the SCWT, TESEN, Rings Test and WAIS–IV Working Memory Index tests are shown in Table 2. Statistically significant differences were found in the SCWT for the word‐colour (*p* = 0.002) and interference (*p* = 0.016) scores, with the CPMP group showing lower performance compared to controls. The CPMP group showed average performance in the word and colour conditions, but below average performance in the word‐colour and interference conditions. In contrast, the control group showed average performance in the colour, word‐colour and interference conditions, and above average performance in the word condition, all adjusted for age and educational level.

**TABLE 2:** Comparison of cognitive performance between the CPMP and control groups on the SCWT, TESEN, Rings Test and WAIS–IV—WMI.

	CPMP (*n* = 30)	CG (*n* = 30)	*Z*	Adjusted *p* (Benjamini–Hochberg)
SCWT—T scores				
Word	52.17 ± 6.50	56.67 ± 7.76	−2.16	0.052
Colour	49.60 ± 8.67	53.20 ± 8.02	−1.79	0.082
Word‐colour	44.00 ± 7.01	51.00 ± 6.25	−4.13	0.002
Interference	43.40 ± 7.49	47.83 ± 5.12	−2.54	0.016
TESEN—Decatypic scores				
Execution	4.73 ± 1.72	7.00 ± 1.17	−4.81	0.002
Speed	4.83 ± 1.80	6.83 ± 1.44	−3.95	0.002
Accuracy	5.70 ± 2.05	6.37 ± 1.63	−1.32	0.187
Rings Test—Decatypic scores	4.90 ± 1.65	6.50 ± 2.00	−3.02	0.002
WAIS—WMI—Composite scores	96.90 ± 12.82	106.97 ± 10.81	−3.05	0.004

*Note:* Values are expressed as mean ± standard deviation. *p* values were obtained using the Mann–Whitney U test and adjusted using the Benjamini–Hochberg correction.

Abbreviations: CG, control group; CPMP, Chronic Primary Musculoskeletal Pain; SCWT, Stroop Colour and Word Test; TESEN, The Pathways Test; WAIS—WMI, Wechsler Adult Intelligence Scale—Working Memory Index.

In the TESEN, significant differences were observed in the execution (*p* = 0.002) and speed (*p* = 0.002) components, with the CPMP group scoring significantly lower than controls. According to the interpretation of decatypic scores adjusted for age and educational level, the CPMP group showed below average performance in both components, whereas the control group performed above average in execution and within the average range in speed. No significant differences were found in the accuracy component, with both groups exhibiting average performance.

The CPMP group also scored significantly lower than controls in the Rings Test (*p* = 0.002), with decatypic scores adjusted for age and educational level indicating below average performance, while the control group scored within the average range.

Regarding the WAIS–IV test, statistically significant group differences were found in the Working Memory Index (*p* = 0.004), with the CPMP group scoring at the lower end of the average range and the control group at the upper end of the average range, adjusted for age and educational level.

Partial correlation analyses, controlling for age, sex, pain intensity and psychological distress, revealed significant negative associations between sleep quality and several cognitive outcomes within the CPMP group (Table 3). Poorer sleep quality was associated with lower performance in TESEN execution (*r* = −0.45; *p* = 0.023), TESEN speed (*r* = −0.41; *p* = 0.038) and the WAIS–IV Working Memory Index (*r* = −0.48; *p* = 0.014).

**TABLE 3:** Partial correlation analysis between sleep quality and cognitive performance in the CPMP group, controlling for age, sex, pain intensity and psychological distress.

	CPMP (*n* = 30)
	PSQI
SCWT—T scores	
Word	−0.22
Colour	−0.39
Word‐colour	−0.16
Interference	−0.10
TESEN—Decatypic scores	
Execution	−0.45*
Speed	−0.41*
Accuracy	−0.33
Rings Test—Decatypic scores	−0.22
WAIS—WMI—Composite scores	−0.48*

*Note:* *p* values were obtained using partial Spearman's correlation analysis.

Abbreviations: CPMP, Chronic Primary Musculoskeletal Pain; PSQI, Pittsburgh Sleep Quality Index; SCWT, Stroop Colour and Word Test; TESEN, The Pathways Test; WAIS—WMI, Wechsler Adult Intelligence Scale—Working Memory Index.

* *p* < 0.05.

Linear regression analyses further confirmed the predictive value of sleep quality for cognitive performance in the CPMP group (Table 4). Poor sleep quality accounted for 40% of the variance in TESEN execution (*β* = −0.54; *p* = 0.012), 37% in TESEN speed (*β* = −0.46; *p* = 0.033) and 35% in the WAIS–IV Working Memory Index (*β* = −0.57; *p* = 0.011).

**TABLE 4:** Regression analysis of sleep quality predicting cognitive performance in the CPMP group, controlling for age, sex, pain intensity and psychological distress.

	CPMP (*n* = 30)			
	*R* ^2^	*β*	*t*	*p*
SCWT—T scores				
Word	0.13	−0.30	−1.25	0.223
Colour	0.16	−0.35	−1.48	0.149
Word‐colour	0.05	−0.21	−0.84	0.411
Interference	0.06	0.01	0.05	0.963
TESEN—Decatypic scores				
Execution	0.40	−0.54	−2.71	0.012
Speed	0.37	−0.46	−2.27	0.033
Accuracy	0.31	−0.35	−1.66	0.110
Rings Test—Decatypic scores	0.06	−0.25	−1.06	0.320
WAIS—WMI—Composite scores	0.35	−0.57	−2.74	0.011

Abbreviations: CPMP, Chronic Primary Musculoskeletal Pain; PSQI, Pittsburgh Sleep Quality Index; SCWT, Stroop Colour and Word Test; TESEN, The Pathways Test; WAIS—WMI, Wechsler Adult Intelligence Scale—Working Memory Index.

## 4 Discussion

Sleep plays a fundamental role in the regulation of cognitive processes, particularly those related to attention, memory and executive functioning (Geiger et al. 2010; Rodrigues and Shigaeff 2022). Disrupted sleep has been consistently associated with cognitive difficulties in both clinical and nonclinical populations, with growing evidence indicating that these effects may be especially pronounced in individuals with chronic pain (Seiger et al. 2024; Sen and Tai 2023; Whale and Gooberman‐Hill 2022). The present study aimed to explore whether sleep quality is associated with cognitive functioning in individuals diagnosed with chronic primary musculoskeletal pain (CPMP). By examining the relationship between sleep quality and cognitive performance across multiple domains, this study contributes to a more nuanced understanding of the neuropsychological profile linked to CPMP and underscores the potential role of sleep as a regulatory factor in this population.

The results of this study indicate that patients with CPMP exhibit poorer cognitive performance compared to healthy controls, particularly in domains associated with executive functioning. Notable differences emerged in selective and sustained attention, processing speed, working memory, planning, problem solving, inhibition and resistance to interference. Participants with CPMP generally performed below the expected range for their age and educational level, whereas those in the control group scored within or above normative expectations. These findings align with previous research highlighting subtle yet consistent impairments in executive domains among individuals with chronic pain (Berryman et al. 2014; Corti et al. 2021; Innes and Sambamoorthi 2020; Zhang et al. 2021). Although these difficulties may not reflect a generalised cognitive deterioration, they can have a noticeable impact on everyday functioning (Baker et al. 2016; Landrø et al. 2013; Mendonça et al. 2024). In the context of chronic pain, executive deficits of this nature are closely linked to difficulties in managing daily demands, including planning and organising activities, regulating behaviour, making adaptive decisions and maintaining social and therapeutic routines. These challenges may ultimately compromise the effectiveness of interventions (Berginström et al. 2024; Higgins et al. 2018; Landrø et al. 2013; Mendonça et al. 2024). Considering their potential functional relevance, these findings highlight the importance of identifying such difficulties in clinical practice and addressing them through targeted support strategies.

Regarding the psychopathological profile, the CPMP group reported significantly higher levels of psychological distress and poorer sleep quality compared with the control group, indicating enduring emotional difficulties and disrupted sleep patterns. With respect to sleep‐related variables, our findings show that poorer sleep quality is significantly associated with reduced cognitive performance in individuals with CPMP, even after controlling for age, sex, pain intensity and psychological distress. Specifically, lower sleep quality was significantly correlated with poorer performance in sustained attention, processing speed and working memory. These associations were further supported by regression analyses, which confirmed that sleep quality significantly predicted performance in these domains. These results are in line with previous evidence suggesting that sleep disturbances can contribute to subtle, yet functionally meaningful, impairments in cognitive functioning among individuals with chronic pain (Bothelius et al. 2019; Chen et al. 2023; Gil‐Ugidos et al. 2021; Wilson et al. 2024). Taken together, these findings highlight the importance of addressing sleep quality in the assessment and treatment of chronic pain, particularly when cognitive difficulties are present. From a clinical perspective, this supports the integration of strategies such as psychoeducation on sleep hygiene, cognitive‐behavioural therapy for insomnia and interdisciplinary approaches that combine pain, sleep and cognitive management (Russin et al. 2025; Seiger et al. 2024; Selvanathan et al. 2021). In addition, routine neuropsychological screening may help identify executive difficulties and guide interventions to improve everyday functioning (Baker et al. 2016; Berginström et al. 2024).

While some previous studies have controlled for variables such as age, sex and psychological distress when examining the relationship between sleep and cognitive functioning, few have also considered pain intensity as a covariate (Curtis et al. 2018; Fang et al. 2019; Ojeda et al. 2018; Wilson et al. 2024). One of the key strengths of the present study is that it addresses this limitation by simultaneously controlling for pain intensity alongside other relevant covariates, thereby enabling a more accurate estimation of the unique contribution of sleep quality to cognitive functioning in individuals with CPMP. Additional strengths include the use of standardised neuropsychological instruments with normative data adjusted for age and education, as well as the inclusion of a healthy control group. These methodological features enhance the validity of the findings and contribute to a more nuanced understanding of the cognitive consequences of poor sleep in chronic pain. Nonetheless, certain limitations must be acknowledged. First, the relatively small sample size may limit the generalisability of the results. Second, the cross‐sectional design restricts the ability to examine how cognitive functioning evolves over time and prevents the establishment of causal relationships between the variables analysed. Moreover, this design does not allow us to determine whether the cognitive differences observed in participants with CPMP are the result of deterioration or reflect pre‐existing individual differences in cognitive functioning. Third, other potentially relevant variables—such as fatigue, the side effects of nonpsychotropic or non‐neurological medications or levels of physical activity—were not specifically controlled for and may have influenced the results. Finally, due to time constraints, only the Working Memory Index from the WAIS‐IV was administered, which limited the assessment of the scale's remaining cognitive domains. These limitations underscore the need for future research to employ longitudinal designs, incorporate broader clinical and neuropsychological assessments, and control for additional factors that may influence cognitive outcomes.

In conclusion, the findings of this study contribute to a deeper understanding of the cognitive difficulties experienced by individuals with chronic primary musculoskeletal pain and highlight the importance of sleep quality as a key factor in this condition. The consistent impairments associated with poor sleep quality, particularly in attention, processing speed, and working memory, underscore the need to incorporate cognitive evaluation into the clinical assessment of chronic pain. Furthermore, the results suggest that poor sleep may not only be a consequence of chronic pain but also a contributing factor to executive dysfunction, providing new insight into the specific role of sleep in this context. Taken together, these findings support the integration of sleep‐focused strategies into both assessment and intervention efforts aimed at improving cognitive and functional outcomes in this population. Future research should explore these associations using longitudinal designs to better understand causality and evaluate the potential benefits of sleep‐targeted interventions on cognitive functioning.

## Author Contributions

This study was designed by A.A.‐M., C.B.‐T. and J.M.M.‐M. Data collection and curation were conducted by A.A.‐M., C.B.‐T. and J.M.M.‐M. Formal analysis was carried out by A.A.‐M., J.M.M.‐M. and C.L.D.‐M. The methodology was developed by A.A.‐M., C.B.‐T., J.M.M.‐M. and C.L.D.‐M. The study was supervised by C.B.‐T., J.M.M.‐M., M.E.G.‐B. and C.L.D.‐M. The original draft was prepared by A.A.‐M., C.B.‐T. and J.M.M.‐M. and edited by M.E.G.‐B. and C.L.D.‐M. All authors have approved the final version of the manuscript and agree to be accountable for all aspects of the work.

## Conflicts of Interest

The authors declare no conflicts of interest.
